# Editorial: Insights in: Psychopathology research

**DOI:** 10.3389/fpsyg.2023.1169631

**Published:** 2023-03-24

**Authors:** Drozdstoy Stoyanov, Diogo Telles Correia, Antoine Bechara, Ofir Turel, Xavier Noel

**Affiliations:** ^1^Department of Psychiatry and Medical Psychology, Plovdiv Medical University, Plovdiv, Bulgaria; ^2^Department of Psychiatry and Medical Psychology, University of Lisbon, Lisbon, Portugal; ^3^Department of Psychology, University of Southern California, Los Angeles, CA, United States; ^4^Information Systems Management, School of Computing and Information Systems, Faculty of Engineering and Information Technology, The University of Melbourne, Melbourne, VIC, Australia; ^5^Laboratoire de Psychologie Médicale et Addictologie, Université Libre de Bruxelles, Brussels, Belgium

**Keywords:** psychopathology, mental disorder (disease), diagnostics, clinical assessment and patient diagnosis, psychodiagnosis methods

Modern psychopathology is going through a period of necessary transition. Traditionally, it has been associated with the historical legacy of Franco-German and Russian classical authors who studied symptoms and syndromes with the precision of clinical descriptions. We can mention the milestone contributions of J. E. D. Esquirol and Jules Baillarger (hallucinations), Karl Ludwig Kahlbaum (catatonia and paraphrenia), W. Mayer-Gross and S. T. Stoianov (oneiroid syndrome), Victor K. Kandinsky (pseudo-hallucinations), Valentin Magnan (delusions), Sergey S. Korsakoff and Carl Wernicke (eponymous syndrome), among many others.

Both J. H. Jackson and H. Ey took cross-sectional and longitudinal approaches to the formation of symptoms and the evolution of syndromes. These led to the concept of dissolution.

It was not until the end of the nineteenth century that psychopathology began to focus on the explanatory mechanisms of mental disorders, which at that time were arguably attributed to degenerative factors from a post-Darwinian evolutionary perspective (Bénédict Morel). In the same period, Emil Kraepelin attempted to introduce what he understood to be a medical or nosological system of mental disorders, sparking controversies over the possibility of producing a universal psychiatric classification. His robust approach was almost immediately confronted by the phenomenological psychopathology of Karl Jaspers, which in practice denied categories in psychopathology and higher-order medical taxonomy. This view is still very influential (Stanghellini and Fuchs, 2013).

As a result, psychopathology in the twentieth century was torn apart by diverse and incompatible conceptual explanations of mental phenomena in health and disease. In order to escape from this impasse, instrumentalist descriptive psychopathology was introduced, where diagnostic procedures and criteria followed clinical ratings and structured interviews. This led to scientific anarchy in the late twentieth century, where “anything goes” and diagnostic systems were under constant debate and revision.

The more recent interventions in neuroscience were not successful in providing an epistemological platform for new psychopathology, as there exist major methodological gaps between psychopathology and neuroscience. The majority of neurobiological studies remained exhaustively focused on state-independent measures, combined as endo-phenotypes. Only a limited number of authors and research groups have targeted clinical states using “symptom” capture paradigms, e.g., verbal acoustic hallucinations (Hugdahl et al., 2022), paranoid delusions, and depressive symptoms (Aryutova et al., 2021).

Despite encouraging advances in molecular neuroscience and functional neuroimaging, especially in the areas of resting-state functional (Stoyanov et al., 2022) and effective connectivity (Kandilarova et al., 2021), the caveats that undermine the production of a meaningful psychiatric nosology remain.

The persistent problem of how to define normal mental functions and classify mental disorders beyond high-level clusters, prototypes, or dimensional diagnosis remains.

This Research Topic is dedicated to new findings in psychopathology research, with the clear understanding that there are many overlaps and shared trans-diagnostic areas of inquiry.

This issue begins with an original paper by Krings et al. that explores the proportion of variance in depressive symptoms that can be explained by processes targeted by behavioral activation. It was concluded that activation, behavioral avoidance, brooding, and anticipatory pleasure are relevant processes to target in order to reduce depressive symptoms, while cognitive control and attentional biases are not (Krings et al.).

The second article in this issue by Marco et al. validates the Multidimensional Existential Meaning Scale in a Spanish-speaking sample. It resulted in an adequate version to assess the three dimensions of meaning in Spanish-speaking participants (Marco et al.).

The third study, by Panov (a), looks at the connection between the degree of dissociation and resistance to therapy in 106 patients with schizophrenia. The author concluded that patients with resistant schizophrenia have a much higher level of dissociation than patients in clinical remission and suggested that resistance to the administered antipsychotics is associated with the presence of high dissociation in the group of resistant patients [Panov (a)].

In the fourth work, also an original study, Panov (b) looks at the relation between functional lateralization and the effect of treatment in patients with schizophrenia. An increased number of patients with cross-dominance left eye dominance was found in patients with schizophrenia [Panov (b)].

The fifth article of this issue, by Vander Zwalmen et al., reviews the current state-of-the-art of cognitive control training as an intervention to reduce vulnerability to depression. Several issues are discussed, such as (i) identifying working mechanisms and potential moderators of CCT; (ii) establishing conditions for the effective use of CCT; and (iii) evaluating possible combinations of CCT with other antidepressant interventions (Vander Zwalmen et al.).

The sixth article included, by Moore et al., consists of a systematic, nationwide survey to assess the relationship between anxiety and depression symptoms and coping skills among Asian American medical students. It was concluded that Asian American students who experience anxiety were more likely to use avoidant or negative coping strategies and that those experiencing depressive symptoms were not more likely to utilize these negative coping strategies (Moore et al.).

The seventh study, by Bodart et al., systematically reviews the physiological reactivity at rest and in response to social or emotional stimuli after a traumatic brain injury. It was concluded that although electrodermal activity responses were frequently reported in patients with traumatic brain injury, other measures did not consistently indicate an impairment in physiological reactivity (Bodart et al.).

The eighth article included, by Carta and Cataudella, consists of a perspective on the historical transformation of adolescence—itself a psychological developmental process embedded in sociocultural history.

The ninth article in this issue is an original study that aims at identifying direct and indirect associations among alexithymia, OCD, cardiac interoception, psychological inflexibility, and self-as-context with DV ASD and depression. The results are discussed in relation to the limitations of the DSM with its categorical focus on protocols for syndromes (Edwards).

The final contribution is a brief research report by Panov (c), which purports to analyze the perceived gender role in patients with schizophrenia, looking for differences between patients with treatment resistance and those in clinical remission. A higher percentage of schizophrenic patients who showed a higher identification with the female gender role was found [Panov (c)].

## Author contributions

DS and DT wrote together the manuscript. AB, OT, and XN edited and approved it. All authors contributed to the article and approved the submitted version.
